# Comparing Projected Fatal Overdose Outcomes and Costs of Strategies to Expand Community-Based Distribution of Naloxone in Rhode Island

**DOI:** 10.1001/jamanetworkopen.2022.41174

**Published:** 2022-11-09

**Authors:** Xiao Zang, Sam E. Bessey, Maxwell S. Krieger, Benjamin D. Hallowell, Jennifer A. Koziol, Shayla Nolen, Czarina N. Behrends, Sean M. Murphy, Alexander Y. Walley, Benjamin P. Linas, Bruce R. Schackman, Brandon D. L. Marshall

**Affiliations:** 1 Department of Epidemiology, School of Public Health, Brown University, Providence, Rhode Island; 2 Rhode Island Department of Health, Providence; 3 Department of Population Health Sciences, Weill Cornell Medical College, New York, New York; 4 Section of General Internal Medicine, Department of Medicine, Boston Medical Center, Boston, Massachusetts; 5 Section of Infectious Diseases, Boston Medical Center, Boston, Massachusetts; 6 Department of Medicine, Boston University School of Medicine, Boston, Massachusetts

## Abstract

**Importance:**

In 2021, the state of Rhode Island distributed 10 000 additional naloxone kits compared with the prior year through partnerships with community-based organizations.

**Objective:**

To compare various strategies to increase naloxone distribution through community-based programs in Rhode Island to identify one most effective and efficient strategy in preventing opioid overdose deaths (OODs).

**Design, Setting, and Participants:**

In this decision analytical model study conducted from January 2016 to December 2022, a spatial microsimulation model with an integrated decision tree was developed and calibrated to compare the outcomes of alternative strategies for distributing 10 000 additional naloxone kits annually among all individuals at risk for opioid overdose in Rhode Island.

**Interventions:**

Distribution of 10 000 additional naloxone kits annually, focusing on people who inject drugs, people who use illicit opioids and stimulants, individuals at various levels of risk for opioid overdose, or people who misuse prescription opioids vs no additional kits (status quo). Two expanded distribution implementation approaches were considered: one consistent with the current spatial distribution patterns for each distribution program type (supply-based approach) and one consistent with the current spatial distribution of individuals in each of the risk groups, assuming that programs could direct the additional kits to new geographic areas if required (demand-based approach).

**Main Outcomes and Measures:**

Witnessed OODs, cost per OOD averted (efficiency), geospatial health inequality measured by the Theil index, and between-group variance for OOD rates.

**Results:**

A total of 63 131 simulated individuals were estimated to be at risk for opioid overdose in Rhode Island based on current population data. With the supply-based approach, prioritizing additional naloxone kits to people who use illicit drugs averted more witnessed OODs by an estimated mean of 18.9% (95% simulation interval [SI], 13.1%-30.7%) annually. Expanded naloxone distribution using the demand-based approach and focusing on people who inject drugs had the best outcomes across all scenarios, averting an estimated mean of 25.3% (95% SI, 13.1%-37.6%) of witnessed OODs annually, at the lowest mean incremental cost of $27 312 per OOD averted. Other strategies were associated with fewer OODs averted at higher costs but showed similar patterns of improved outcomes and lower unit costs if kits could be reallocated to areas with greater need. The demand-based approach reduced geospatial inequality in OOD rates in all scenarios compared with the supply-based approach and status quo.

**Conclusions and Relevance:**

In this decision analytical model study, variations in the effectiveness, efficiency, and health inequality of the different naloxone distribution expansion strategies and approaches were identified. Future efforts should be prioritized for people at highest risk for overdose (those who inject drugs or use illicit drugs) and redirected toward areas with the greatest need. These findings may inform future naloxone distribution priority settings.

## Key Points

**Question:**

What community-based naloxone distribution strategies would be the most effective and efficient in preventing opioid overdose deaths?

**Findings:**

In this decision analytical model study evaluating the distribution of 10 000 additional naloxone kits annually in Rhode Island, the strategy focusing on distribution of naloxone according to geographic need to people who inject drugs resulted in the best outcomes at the lowest cost, averting an estimated 25.3% of opioid overdose deaths at an incremental cost of $27 312 per opioid overdose death averted.

**Meaning:**

This study suggests that expanding naloxone distribution to people at highest risk for opioid overdose should be prioritized and that redirecting spatial distribution of naloxone to areas with the greatest need will improve both effectiveness and efficiency and reduce geospatial health inequality.

## Teaser

This decision analytical model study compares the fatal overdose outcomes and costs associated with various strategies to increase naloxone distribution through community-based programs in Rhode Island.

## Introduction

The United States recorded nearly 71 000 drug overdose deaths in 2019.^1^ This number increased by approximately 30% in 2020 and another 15% in 2021, such that at this time, the Centers for Disease Control and Prevention (CDC) estimate more than 100 000 overdose deaths occur annually.^2^ In Rhode Island, drug overdose deaths increased by nearly 25% in 2020 compared with 2019 and 14% in 2021 compared with 2020, in contrast with a decrease in overdose deaths from 2016 to 2019.^3^ The increase in drug overdose deaths during the COVID-19 era can be attributed to social and physical isolation, decreased access to substance use treatment and harm reduction services, and the increasing presence of fentanyl, a rapidly acting synthetic opioid with a narrow response window, in the illicit drug supply, which accounted for 76% of drug overdose deaths in Rhode Island during the COVID-19 era.^4^

Naloxone is an opioid antagonist medication that is safe and effective in reversing an opioid overdose.^5,6,7^ Naloxone distribution to laypersons plays an important role in reducing opioid overdose deaths (OODs).^8^ Rhode Island has among the highest rates of per capita naloxone distribution in the US.^9,10,11^ Rhode Island enacted overdose-related laws to remove barriers to naloxone access and rescue, including naloxone dispensation under standing orders, pharmacy-based naloxone access, and Good Samaritan laws.^12,13^ As of 2021, there were more than 15 agencies providing opioid overdose education and naloxone distribution (OEND) programs across Rhode Island,^14^ each servicing distinct population groups at different risks for opioid overdose in different communities. Naloxone distribution through each of these programs may require different levels of resources and may have different levels of effectiveness in reducing OODs.

Naloxone is a cost-effective intervention to save lives,^15,16,17^ but evidence is needed to guide future priority setting for expanded naloxone distribution across population groups and geographic areas to optimize the allocation of available resources and improve health equity. In 2021, in response to the escalating drug overdose crisis during the COVID-19 pandemic and the increasing demand for naloxone, the Rhode Island Department of Health (RIDOH) initiated a “10,000 Chances Project,” which aimed to distribute more than 10 000 additional naloxone kits (2 doses per kit) through partnerships with local, community-based naloxone distribution organizations.^18^ A prior study found geographic variations in OODs in Rhode Island and also found that patterns of naloxone distribution from OEND programs did not fully correspond to the burden of OODs.^14^ This study aims to assess and compare the effectiveness, efficiency, and geospatial health equity of alternative naloxone distribution expansion strategies through community-based programs in Rhode Island.

## Methods

In this decision analytical model study conducted from January 2016 to December 2022, we developed a microsimulation model with an integrated decision tree model in R, version 4.0.5 (R Group for Statistical Computing)^19^ to estimate the outcomes and costs of distributing 10 000 additional naloxone kits each year (on the basis of 2019 naloxone distribution) through different distribution strategies in Rhode Island over a 3-year time horizon. We also created an online web tool using Vue to visualize our model and display scenario results.^20^ This is a simulation modeling study that generates simulated individuals and integrates data from the literature to project likely outcomes. The work was deemed to not be human participants research by the Brown University and Boston University Medical Campus institutional review boards.

### Model Description

**Figure 1.**
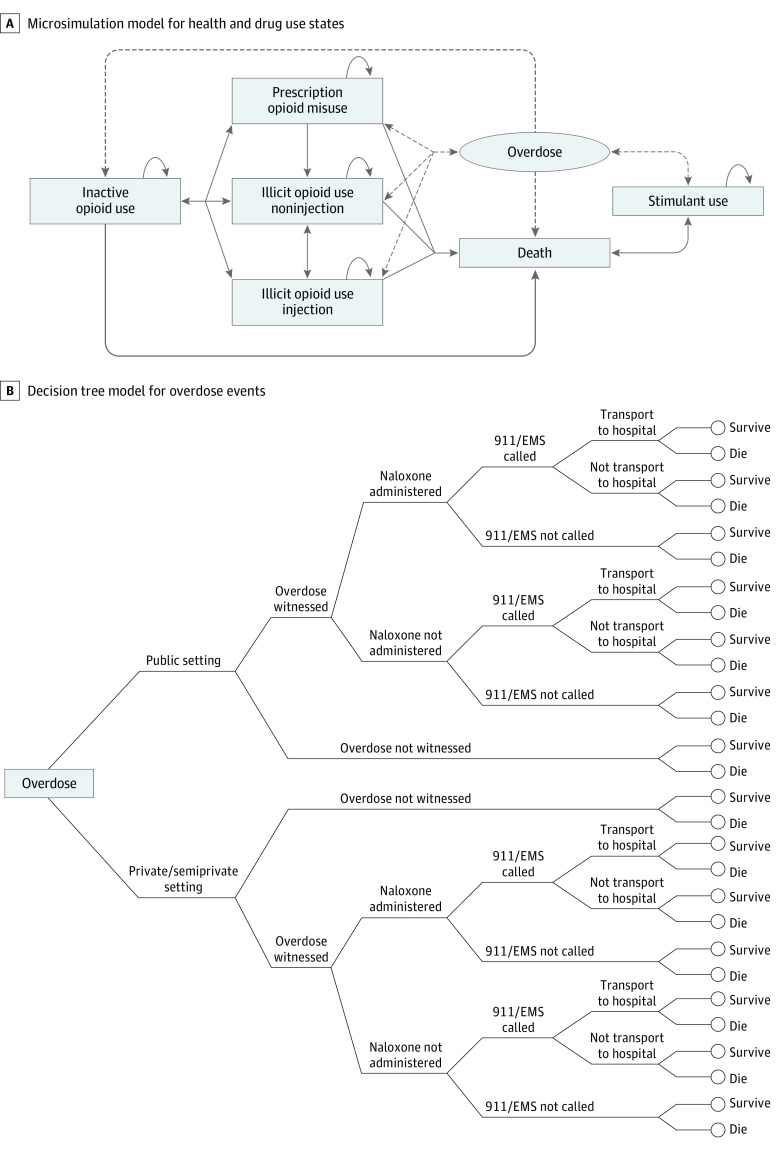
Model Structure Diagram A, Microsimulation model for health and drug use states. The solid lines indicate transitions among health states, whereas the dashed lines indicate transitions between health states and overdose. B, Decision tree model for overdose events. EMS indicates emergency medical services.

We developed an individual-based microsimulation model (Figure 1A) with a monthly cycle length to simulate transitions between health states and drug use patterns and to project the number of overdose events in each month. The model simulates the progression of a virtual cohort representing all individuals in Rhode Island who are at risk for opioid overdose. We characterized simulated individuals by sex (male or female), age, race and ethnicity (self-identified in census data: Black or African American, Hispanic or Latino, non-Hispanic White, and other [any racial or ethnic category that was not Hispanic or Latino, non-Hispanic White, or Non-Hispanic Black]), and city or town of residence to account for different prevalences of drug use across demographic groups. We also stratified the population by patterns of drug use (defined as exclusive prescription opioid misuse, any illicit opioid use [heroin or illicit synthetic opioid], inactive opioid use, and exclusive illicit stimulant use without intended opioid use), route of administration (injection or noninjection), prior opioid overdose history (yes or no), and fentanyl exposure (intentional use of fentanyl or fentanyl-contaminated drugs; yes or no) to capture the major factors associated with an individual’s overdose risk.^17^ Because naloxone is effective in reversing overdoses caused only by opioids, we modeled opioid overdose only. We considered elevated risks for overdose for those who were exposed to fentanyl,^21^ those who use opioids through injection,^22,23^ those with a history of opioid overdose,^24^ and during the first month out of the inactive opioid use state.^21^ We included the exclusive stimulant use state to account for increasing presence of fentanyl in cocaine and methamphetamine and the resulting opioid-related overdoses occurring within this group.^25^

We then integrated a decision tree model (Figure 1B) to assess the potential pathway and consequence of each overdose event. We characterized each overdose event by attributes associated with the probability of overdose death, including the setting of overdose (public vs private or semiprivate), whether the overdose was witnessed, whether naloxone was administered (among witnessed overdoses), whether emergency medical services were dispatched as a result of a witness calling 911, and whether the simulated overdosing individual was transported to the hospital for emergency department care. If the simulated individual survived, this individual then returned to the microsimulation into either inactive opioid use^26^ or the same active drug use state as prior to overdose, but with an overdose history.

We established a naloxone availability algorithm to estimate the probability of naloxone being available during an overdose event, assuming that the probability that the medication was administered during a witnessed overdose in a city or town is a nonlinear function of the number of naloxone kits in circulation and the number of individuals at risk for opioid overdose in the same jurisdiction (eAppendix in the Supplement).^27^ We included naloxone kits distributed through OEND programs and pharmacies in this algorithm. We estimated that pharmacies are less effective than OEND programs at increasing naloxone availability among people at risk for opioid overdose, based on British Columbia’s Take-Home Naloxone program data.^28^ The naloxone availability algorithm assigned the new naloxone kits distributed to become available each month and withdrew 6.5% (1 per 15.5 months) of existing naloxone kits from circulation each month owing to expiration or loss, based on an analysis of the New York City Department of Health and Mental Hygiene data (Andrew Trinidad, MPH, email, February 9, 2021).

We derived population sizes and demographic characteristics for each city or town in Rhode Island from the US Census Bureau and estimated the prevalence of drug use (of different types) among each sociodemographic subgroup based on data from the National Survey on Drug Use and Health.^29^ Information about how many naloxone kits distributed by OEND programs were received by residents of different cities or towns was derived from data collected by RIDOH from programs.^14^ The annual number of OODs was aggregated at the city or town level according to the residence of the deceased individual based on data from the RIDOH Office of State Medical Examiners. Other parameters were primarily obtained from published literature and are presented in eTable 1 in the Supplement.

### Statistical Analysis

#### Model Calibration and Validation

We calibrated our model using a random calibration approach to match 3 sets of observed state-level target data between 2016 and 2019^3^: (1) annual number of OODs, (2) percentage of OODs involving fentanyl, and (3) annual number of emergency department visits associated with opioid overdose. We used a Latin hypercube sampling method^30^ to draw 1 000 000 random samples of parameters from their prior ranges to inform the model and retained 500 subsets providing the best fit for subsequent analysis. This method allowed us to capture both the parameter uncertainty and stochasticity inherent in a microsimulation model. All model results are presented as mean estimates and 95% simulation intervals (SIs). For model validation, we further compared our projections for the number of OODs in each city or town with surveillance data. More details regarding model calibration and validation can be found in the eAppendix in the Supplement.

#### Modeling Scenarios

We compared the projected outcomes of 4 strategies focusing on different populations for distributing 10 000 additional naloxone kits annually: (1) people who inject illicit opioids or stimulants (people who inject drugs) (eg, syringe service programs), (2) people who use illicit opioids and/or stimulants through injection or noninjection (people who use illicit drugs) (eg, street outreach programs), (3) all study populations at various levels of risk of opioid overdose (all risk groups) (eg, distribution through community events), and (4) people who exclusively misuse prescription opioids (eg, opioid use disorder treatment centers). For all scenarios, we considered 2 expanded distribution implementation approaches: (1) consistent with the current spatial distribution patterns of naloxone distribution using historical data for the proportions of kits received by residents of each city or town from the exemplary programs operating in Rhode Island, according to RIDOH data (supply-based approach) (eAppendix in the Supplement), and (2) consistent with the current spatial distribution of individuals in each of the risk groups, assuming programs could direct the additional kits to new geographic areas if required (demand-based approach). We assumed that all naloxone expansion strategies would be sustained for 3 years. Outcomes from these naloxone distribution expansion scenarios were compared with a status quo scenario that maintained the 2019 levels of naloxone distribution. For all scenarios, we assumed that the number of naloxone kits distributed by pharmacies remained unchanged from 2019.

#### Costs and Outcomes

We estimated total costs (in 2019 US dollars) associated with distributing 10 000 additional kits annually under the different scenarios that included both naloxone material cost and cost of distribution. The material cost estimate was based on the proportions of different types of naloxone kits (intramuscular vs intranasal) distributed in 2020 and 2021 and the purchasing price to RIDOH for each kit type. We estimated the unit cost for distributing kits for each evaluated strategy based on a costing analysis of OEND programs^31^ (eAppendix in the Supplement). The effectiveness of each strategy was measured as the number of OODs averted, and the efficiency of the strategy was measured as the cost per OOD averted.

We also assessed geospatial health inequality for each strategy by comparing deviation of OOD rates across cities or towns from the state mean under each scenario during the last year of the evaluation period. We used the Theil index and between-group variance^32^ as summary measures of geospatial inequality. For both measures, larger values indicate greater inequality (eAppendix in the Supplement).

## Results

**Figure 2.**
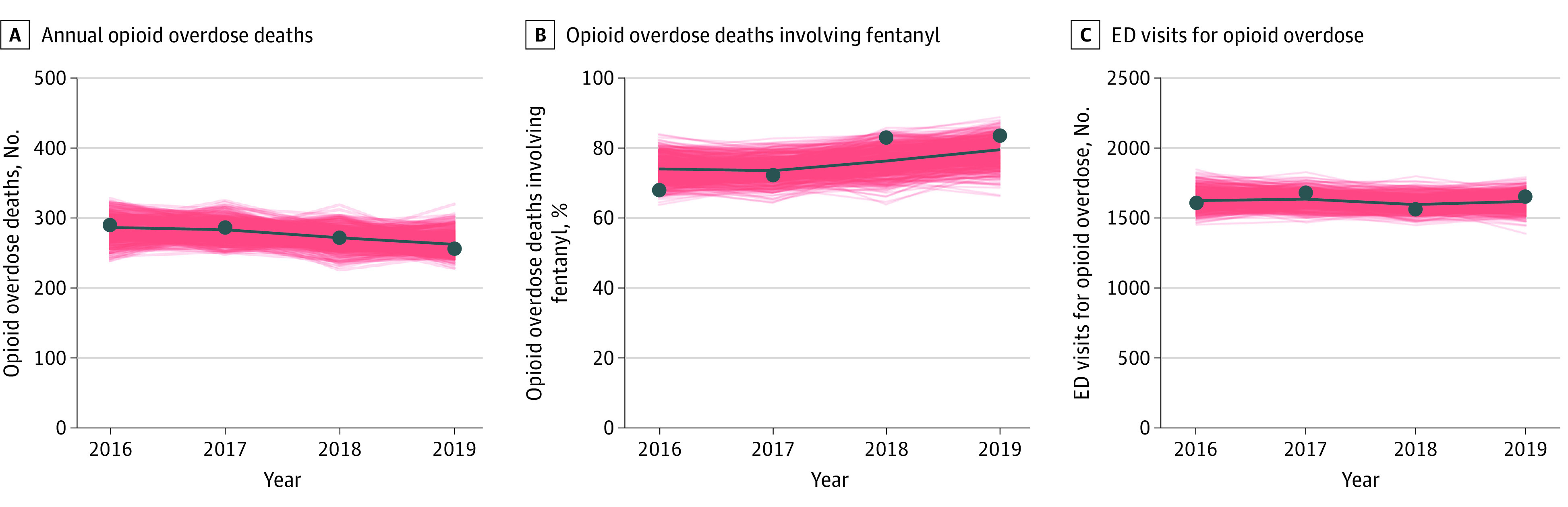
Model Calibration to Observed Opioid Overdose–Related Targets in Rhode Island The red shaded areas indicate model results from 500 calibrated parameter sets. Calibration targets are based on Rhode Island Department of Health data. ED indicates emergency department.

We estimated a total of 63 131 individuals who were at risk for opioid overdose in Rhode Island based on current population data. After calibration, our model demonstrated excellent fit to the selected targets associated with opioid overdoses, with a mean percentage deviation of 0.05 (Figure 2). The reported number of OODs in each city or town all fell within the 95% SI of the model estimates except for 1 municipality (eFigure 4 in the Supplement). Maintaining the 2019 level of naloxone distribution (status quo), we estimated a total of 756 OODs (95% SI, 664-859) (among which 349 [95% SI, 221-519] were witnessed) during the subsequent 3 years, corresponding to a mean of 252 (95% SI, 221-286) OODs per annum (compared with 256 observed in 2019).

**Figure 3.**
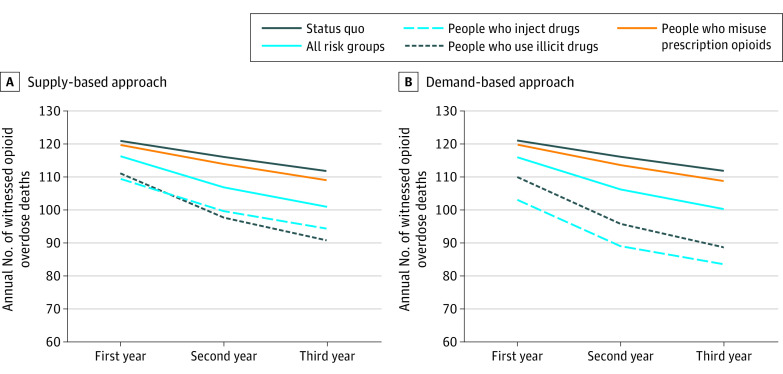
Estimated Annual Number of Witnessed Opioid Overdose Deaths in Each Naloxone Distribution Expansion Scenario Results are based on the mean estimates from 500 calibrated parameter sets.

Figure 3 shows the estimated annual number of witnessed OODs in each scenario during the 3-year evaluation period. Distributing 10 000 additional naloxone kits and focusing on people who inject drugs averted an estimated mean of 45.5 OODs (95% SI, 21.0-82.5) (relative reduction, 13.0% [95% SI, 7.1%-19.8%]) during the 3-year period using the supply-based approach and 73.4 OODs (95% SI, 34.0-128.0) (relative reduction, 21.1% [95% SI, 12.8%-29.7%]) using the demand-based approach. A strategy prioritizing people who use illicit drugs (opioids and/or stimulants) averted an estimated mean of 49.5 OODs (95% SI, 20.0-94.1) (relative reduction, 14.0% [95% SI, 7.7%-21.7%]) during the 3-year period using the supply-based approach and 54.7 OODs (95% SI, 23.0-101.5) (relative reduction, 15.5% [95% SI, 8.6%-23.7%]) using the demand-based approach. Expanding naloxone distribution to all risk groups would averted an estimated mean of 24.8 OODs (95% SI, 7.5-47.5) (relative reduction, 7.1% [95% SI, 2.7%-12.0%]) during the 3-year period using the supply-based approach and 26.5 OODs (95% SI, 10.0-51.5) (relative reduction, 7.6% [95% SI, 3.5%-11.9%]) using the demand-based approach. Distribution of additional naloxone kits targeting people who misuse prescription opioids was the least effective at averting OODs, by a mean of 6.1 (95% SI, 0-19.0) (relative reduction, 1.8% [95% SI, 0%-4.6%]) during the 3-year period using the supply-based approach and a mean of 6.8 OODs (95% SI, 0-19.5) (relative reduction, 1.9% [0%-5.0%]) using the demand-based approach.

Across all expansion scenarios, we found that the greatest health benefits were achieved in the last year of the evaluation period owing to the cumulative increase in the number of naloxone kits in circulation (Figure 3). The Table presents results in the last year of the evaluation period. With the supply-based approach, prioritizing additional naloxone kits for people who use illicit drugs was projected to avert more witnessed OODs by 18.9% (95% SI, 13.1%-30.7%) annually. We estimated that expanding distribution to people who inject drugs would likely avert 25.3% (95% SI, 13.1%-37.6%) of the annual number of OODs with the demand-based approach.

**Table.** Estimated Outcomes and Costs for Strategies to Increase Naloxone Distribution in Rhode Island Through Community-Based Programs in the Last Year of the 2020-2022 Evaluation Period

Strategy	Supply-based approach				Demand-based approach			
	Witnessed OODs averted, No. (%)^a^	Cost per OOD averted, $	Theil index	Between-group variance	Witnessed OODs averted, No. (%)^a^	Cost per OOD averted, $	Theil index	Between-group variance
Status quo	NA	NA	2.53	3.63	NA	NA	2.53	3.63
Expanded distribution target population								
People who inject drugs	17.5 (15.6)	44 185	3.48	4.43	28.3 (25.3)	27 312	2.48	2.81
People who use illicit drugs	21.1 (18.9)	78 364	3.89	4.76	23.2 (20.8)	71 104	2.53	2.96
All risk groups	10.9 (9.7)	200 147	3.31	4.29	11.5 (10.3)	188 345	2.45	3.18
People who misuse prescription opioids	2.8 (2.5)	610 992	2.53	3.56	3.1 (2.7)	557 655	2.53	3.56

Abbreviations: NA, not applicable; OOD, opioid overdose death.

^a^ Compared with the status quo scenario; the percentages represent relative reduction in witnessed OODs. Results are averaged from 500 calibrated parameter sets. The between-group variance and Theil index both measure the level of geospatial health inequality based on difference in rates of OOD across cities or towns, where larger values indicate greater inequality. Detailed descriptions of these 2 measures are available in the eAppendix in the Supplement.

Based on results in the last year of evaluation, expanding naloxone distribution focusing on people who inject drugs was associated with lower mean costs per OOD averted of $44 185 under the supply-based approach and $27 312 under the demand-based approach, in contrast to the other 3 strategies with substantially higher unit costs (Table).

Although geospatial inequality (based on the rates of OODs across cities or towns) increased from the status quo under the supply-based approach, the demand-based approach was associated with lower levels of inequality across all expansion scenarios (Table). For example, in the expansion scenario focusing on people who inject drugs, the between-group variance for the annual rate of OODs per 100 000 people was 4.43 with the supply-based approach vs 2.81 with the demand-based approach compared with 3.63 in the status quo.

## Discussion

We developed a microsimulation model calibrated to opioid overdose surveillance data to evaluate and compare the effectiveness, efficiency, and geospatial health equity of alternative strategies to expand naloxone distribution in Rhode Island. This study highlights the need for expanded naloxone distribution efforts to reduce OODs, while the strategies and approaches through which naloxone is distributed are critical to the effectiveness and efficiency of each naloxone kit distributed. Distributing 10 000 additional naloxone kits based on historical geospatial naloxone distribution patterns produced the greatest benefits when kits were prioritized to people who use illicit opioids and/or stimulants. Distributing additional naloxone kits based on the estimated geospatial distribution of the population at risk was associated with more OODs averted and reduced geospatial inequality in OOD rates. Assuming this approach, prioritizing populations who inject drugs dominated, reducing witnessed OODs by more than 25% compared with the status quo. In all strategies, the cost per OOD death averted was significantly lower than the Rhode Island Office of Regulatory Reform’s accepted threshold for cost per life saved ($9.1 million).^33^

Our study findings are consistent with assessments by the American Society of Addiction Medicine,^34^ the CDC,^35^ and previous research studies^36,37^ that have recommended developing more effective naloxone distribution strategies to reach the highest-need locations and populations. An analysis by the CDC of naloxone dispensing showed that rural counties were more likely to be underserved,^9^ and a prior study also identified disparities in naloxone access between urban and nonurban regions in Rhode Island.^14^ Although this analysis was conducted in Rhode Island, it provides evidence about the effectiveness and cost of alternative approaches to address these disparities that has implications for naloxone distribution efforts and priority setting in other states in the US. For example, the consistent advantage associated with a demand-based approach suggests that collecting data on the demand for naloxone and using the data to guide naloxone distribution may improve overdose reduction efforts. Nevertheless, there are other existing barriers and challenges in naloxone distribution that may potentially result in missed opportunities to decrease opioid overdose fatalities. For example, racial and ethnic minority groups and those experiencing homelessness have been found to be less likely to have access to naloxone.^38,39^ Additional research and improved surveillance data stratified by subpopulation (eg, race and ethnicity) and region are needed to address these barriers and facilitate the implementation of the recommended strategies to maximize the population health impact of naloxone distribution.

The set of strategies for naloxone distribution examined in this study should not be considered exhaustive. Other distribution programs also play important roles, including naloxone distribution from emergency departments as well as other social service programs.^40^ Novel strategies, such as vending machines for dispensing naloxone kits, are being implemented in Rhode Island and in other communities across the US.^41^ Naloxone vending machines are a low-barrier option for underserved populations and areas, having the potential to be a practical and inexpensive method to enhance widespread access to naloxone. Examining these different strategies can be particularly valuable for future naloxone distribution efforts in Rhode Island. In March 2022, the state reached a settlement with Teva Pharmaceuticals, who will supply 50 000 kits of naloxone, which is more than 5 times the number of kits distributed by Rhode Island OEND programs in 2019, each year for the next 10 years.^42^

One critical determinant for the effectiveness of naloxone distribution is the proportion of overdose events that are witnessed. In the midst of the social and physical isolation that has increased during the COVID-19 pandemic and the underlying stigmatization of drug use, it is crucial to find more ways to reduce events in which drug use occurs alone, such as supervised consumption sites, which were recently authorized by the state of Rhode Island and have the potential to reach individuals with the highest risk for overdose.^43,44^ Future studies should consider the potential effectiveness and cost of additional investments in these programs to expand their distribution capacities.

There is clearly a need for a robust, multifaceted approach to the opioid overdose epidemic beyond providing take-home naloxone kits.^45^ Although increasing naloxone access is a critical component of the public health response that provides substantial short-term benefits, it alone is insufficient to address the persistent opioid overdose crisis in the US. Naloxone distribution should be integrated with other effective long-term strategies for preventing opioid overdose,^46^ such as academic detailing, fentanyl testing strip distribution, over-the-counter naloxone, and increasing access to treatment for opioid use disorder.^35^ Evaluating all these interventions in combination under 1 analytical framework (eg, using a simulation model) may help build a more holistic approach for comprehensive overdose prevention efforts, which may lead to more efficient resource allocation.^47^

### Limitations

This study has several limitations. First, although the 10,000 Chances Project was initiated as a response to drug overdose escalation during the COVID-19 pandemic, we chose the pre–COVID-19 period to be our baseline for calibration and evaluation owing to limited data on the detailed impact of COVID-19 on the opioid overdose epidemic (eg, increased at-risk population, increased presence of fentanyl in drug supplies, interruptions to other health care services, or a combination of these factors). Our estimates for the effectiveness of expanded naloxone distribution should otherwise be considered conservative if these factors are considered. Second, geospatial estimates of target populations were based on extrapolations from the National Survey on Drug Use and Health (eAppendix in the Supplement), in the absence of local data. Results might be different if other methods (eg, capture-recapture^48^) were used to estimate the target population size by city or town. Third, although we built the model to reflect the opioid overdose epidemic and naloxone distribution in each city or town, our model was not calibrated to targets at the city or town level owing to computational complexity and unavailable jurisdiction-specific target data. Most model parameters, except the initial population estimates and access to naloxone from OEND programs, were uniformly defined and calibrated at the state level. Meanwhile, geographic differences in the density of pharmacies and in naloxone access from pharmacies were not modeled owing to lack of available data on the recipients of naloxone dispensed at pharmacies. However, the model validation results indicated that our projections were credible at the local level. Fourth, the model’s structural design may have some limitations; we did not explicitly model treatment of opioid use disorder but instead modeled inactive opioid use as a proxy, which might result in an overestimation of the overdose risk for the population that was receiving treatment but did not cease opioid use. In the patterns of drug use that we modeled, we included the major ones found in fatal opioid overdoses in Rhode Island, including fentanyl-contaminated cocaine and methamphetamine, but did not include less commonly documented patterns, such as fentanyl-contaminated benzodiazepines or prescription stimulants.^49^ Fifth, in the demand-based scenario, we implicitly assumed that the additional kits could be directed to new geographic areas even when there were no existing programs serving the target population living in these areas. The feasibility and additional costs associated with expanding existing programs to new areas or opening new programs were not explicitly considered in this study.

## Conclusions

In this decision analytical model study, our study findings suggest that naloxone rescue kits could be most cost-effective and life-saving when distributed via syringe service programs, street outreach, and other programs serving people who inject drugs. Efforts to expand naloxone distribution according to the greatest geospatial need could yield additional public health benefits while reducing geospatial health inequality.

## Notes

Supplement.
eAppendix.
**eFigure 1.** Drug Use State Stratification **eFigure 2.** Map of Cities/Towns in Rhode Island **eTable 1.** Past Year Opioid Misuse and Exclusive Stimulant Use Based on the NSDUH Data (Before Adjustment) **eTable 2.** Variable Names Used in the NSDUH Data Derivation **eTable 3.** Study Population Parameters **eTable 4.** Risk of Opioid Overdose for Each Opioid Use Health State **eTable 5.** Monthly Health State Transition Probability Matrix **eTable 6.** Background Age-Stratified Mortality Rate Excluding Death Due to Overdose **eTable 7.** Decision Tree Parameters **eFigure 3.** Results of Preliminary Model Calibration to Observed Fatal and Non-Fatal Overdose Setting Data **eTable 8.** Preliminary Calibration Parameters and Targets **eTable 9.** Calibrated Parameters and Values **eFigure 4.** Model Validation to the City/Town-Level Number of Opioid Overdose Deaths in 2019 **eTable 10.** Cost of Naloxone Kit **eTable 11.** Overdose Education and Naloxone Distribution (OEND) Average Costs **eTable 12.** Summary Inequality Measures **eTable 13.** Proportions of Kits Received by Residents of Each City/Town From Each Exemplary Program Type, According to Rhode Island Department of Health Data
eReferences.

## References

[1] Hedegaard H, Miniño AM, Warner M. Drug overdose deaths in the United States, 1999-2019. NCHS Data Brief, no 394. National Center for Health Statistics. December 2020. Accessed December 28, 2020. https://www.cdc.gov/nchs/data/databriefs/db394-H.pdf

[2] National Vital Center for Health Statistics. Provisional drug overdose death counts. 2022. Accessed May 24, 2022. https://www.cdc.gov/nchs/nvss/vsrr/drug-overdose-data.htm

[3] Prevent Overdose RI. Overdose death data, Rhode Island. Accessed August 19, 2021. https://preventoverdoseri.org/overdose-deaths/

[4] Macmadu A, Batthala S, Correia Gabel AM, . Comparison of characteristics of deaths from drug overdose before vs during the COVID-19 pandemic in Rhode Island. JAMA Netw Open. 2021;4(9):e2125538. doi:10.1001/jamanetworkopen.2021.25538 34533569 PMC8449276

[5] US Department of Health and Human Services. Opioid abuse in the United States and Department of Health and Human Services actions to address opioid-drug–related overdoses and deaths. J Pain Palliat Care Pharmacother. 2015;29(2):133-139. doi:10.3109/15360288.2015.1037530 26095483

[6] McDonald R, Strang J. Are take-home naloxone programmes effective? systematic review utilizing application of the Bradford Hill criteria. Addiction. 2016;111(7):1177-1187. doi:10.1111/add.13326 27028542 PMC5071734

[7] Walley AY, Xuan Z, Hackman HH, . Opioid overdose rates and implementation of overdose education and nasal naloxone distribution in Massachusetts: interrupted time series analysis. BMJ. 2013;346:f174. doi:10.1136/bmj.f174 23372174 PMC4688551

[8] Fairbairn N, Coffin PO, Walley AY. Naloxone for heroin, prescription opioid, and illicitly made fentanyl overdoses: challenges and innovations responding to a dynamic epidemic. Int J Drug Policy. 2017;46:172-179. doi:10.1016/j.drugpo.2017.06.005 28687187 PMC5783633

[9] Guy GP Jr, Haegerich TM, Evans ME, Losby JL, Young R, Jones CM. Vital signs: pharmacy-based naloxone dispensing—United States, 2012–2018. MMWR Morb Mortal Wkly Rep. 2019;68(31):679-686. doi:10.15585/mmwr.mm6831e1 31393863 PMC6687198

[10] Lambdin BH, Zibbell J, Wheeler E, Kral AH. Identifying gaps in the implementation of naloxone programs for laypersons in the United States. Int J Drug Policy. 2018;52:52-55. doi:10.1016/j.drugpo.2017.11.017 29232604

[11] Lambdin BH, Davis CS, Wheeler E, Tueller S, Kral AH. Naloxone laws facilitate the establishment of overdose education and naloxone distribution programs in the United States. Drug Alcohol Depend. 2018;188:370-376. doi:10.1016/j.drugalcdep.2018.04.004 29776688

[12] McClellan C, Lambdin BH, Ali MM, . Opioid-overdose laws association with opioid use and overdose mortality. Addict Behav. 2018;86:90-95. doi:10.1016/j.addbeh.2018.03.014 29610001

[13] Pollini RA, Joyce R, Ozga-Hess JE, Xuan Z, Green TC, Walley AY. Assessing pharmacy-based naloxone access using an innovative purchase trial methodology. J Am Pharm Assoc (2003). 2020;60(6):853-860. doi:10.1016/j.japh.2020.05.016 32651116 PMC7655699

[14] Zang X, Macmadu A, Krieger MS, . Targeting community-based naloxone distribution using opioid overdose death rates: a descriptive analysis of naloxone rescue kits and opioid overdose deaths in Massachusetts and Rhode Island. Int J Drug Policy. 2021;98:103435. doi:10.1016/j.drugpo.2021.103435 34482264 PMC8671216

[15] Coffin PO, Sullivan SD. Cost-effectiveness of distributing naloxone to heroin users for lay overdose reversal. Ann Intern Med. 2013;158(1):1-9. doi:10.7326/0003-4819-158-1-201301010-00003 23277895

[16] Townsend T, Blostein F, Doan T, Madson-Olson S, Galecki P, Hutton DW. Cost-effectiveness analysis of alternative naloxone distribution strategies: first responder and lay distribution in the United States. Int J Drug Policy. 2020;75:102536. doi:10.1016/j.drugpo.2019.07.031 31439388

[17] Acharya M, Chopra D, Hayes CJ, Teeter B, Martin BC. Cost-effectiveness of intranasal naloxone distribution to high-risk prescription opioid users. Value Health. 2020;23(4):451-460. doi:10.1016/j.jval.2019.12.002 32327162

[18] State of Rhode Island Department of Behavioral Healthcare, Developmental Disabilities & Hospitals. Statewide project launches in response to rise in overdose deaths during COVID-19. January 7, 2021. Accessed October 5, 2021. https://bhddh.ri.gov/sites/g/files/xkgbur411/files/documents/Press-Release-10000-Chances.pdf

[19] R Core Team. R: a language and environment for statistical computing. R Foundation for Statistical Computing. 2021. Accessed December 21, 2021. https://www.R-project.org/

[20] PROFOUND (Prevention and Rescue of Fentanyl and Other Opioid Overdoses Using Optimized Naloxone Distribution Strategies) is a five-year study aiming to develop a tool to optimize naloxone distribution strategies. PROFOUND. Accessed October 3, 2022. https://profoundmodel.org/

[21] Merrall EL, Kariminia A, Binswanger IA, . Meta-analysis of drug-related deaths soon after release from prison. Addiction. 2010;105(9):1545-1554. doi:10.1111/j.1360-0443.2010.02990.x 20579009 PMC2955973

[22] Kerr T, Fairbairn N, Tyndall M, . Predictors of non-fatal overdose among a cohort of polysubstance-using injection drug users. Drug Alcohol Depend. 2007;87(1):39-45. doi:10.1016/j.drugalcdep.2006.07.009 16959438

[23] Brugal MT, Barrio G, De LF, Regidor E, Royuela L, Suelves JM. Factors associated with non-fatal heroin overdose: assessing the effect of frequency and route of heroin administration. Addiction. 2002;97(3):319-327. doi:10.1046/j.1360-0443.2002.00058.x 11964108

[24] Darke S, Williamson A, Ross J, Mills KL, Havard A, Teesson M. Patterns of nonfatal heroin overdose over a 3-year period: findings from the Australian Treatment Outcome Study. J Urban Health. 2007;84(2):283-291. doi:10.1007/s11524-006-9156-0 17265131 PMC2231629

[25] LaRue L, Twillman RK, Dawson E, . Rate of fentanyl positivity among urine drug test results positive for cocaine or methamphetamine. JAMA Netw Open. 2019;2(4):e192851. doi:10.1001/jamanetworkopen.2019.2851 31026029 PMC6487565

[26] Langabeer J, Champagne-Langabeer T, Luber SD, . Outreach to people who survive opioid overdose: linkage and retention in treatment. J Subst Abuse Treat. 2020;111:11-15. doi:10.1016/j.jsat.2019.12.008 32087833

[27] Irvine MA, Oller D, Boggis J, . Estimating naloxone need in the USA across fentanyl, heroin, and prescription opioid epidemics: a modelling study. Lancet Public Health. 2022;7(3):e210-e218. doi:10.1016/S2468-2667(21)00304-2 35151372 PMC10937095

[28] Moustaqim-Barrette A, Papamihali K, Mamdani Z, Williams S, Buxton JA. Accessing take-home naloxone in British Columbia and the role of community pharmacies: results from the analysis of administrative data. PLoS One. 2020;15(9):e0238618. doi:10.1371/journal.pone.0238618 32915834 PMC7485887

[29] Substance Abuse and Mental Health Data Archive. National Survey on Drug Use and Health: 2-year RDAS (2018 to 2019). Accessed March 5, 2021. https://rdas.samhsa.gov/#/survey/NSDUH-2018-2019-RD02YR

[30] Helton JC, Davis FJ. Latin hypercube sampling and the propagation of uncertainty in analyses of complex systems. Reliab Eng Syst Saf. 2003;81(1):23-69. doi:10.1016/S0951-8320(03)00058-9

[31] Behrends CN, Gutkind S, Winkelstein E, . Costs of opioid overdose education and naloxone distribution in New York City. Subst Abus. 2022;43(1):692-698. doi:10.1080/08897077.2021.1986877 34666633 PMC9048167

[32] Hosseinpoor AR, Nambiar D, Schlotheuber A, Reidpath D, Ross Z. Health Equity Assessment Toolkit (HEAT): software for exploring and comparing health inequalities in countries. BMC Med Res Methodol. 2016;16(1):141. doi:10.1186/s12874-016-0229-9 27760520 PMC5069829

[33] Rhode Island Office of Regulatory Reform. Analyzing regulatory benefits and costs: a guide for Rhode Island executive agencies. September 2015. Accessed July 22, 2021. https://rigov-policies.s3.amazonaws.com/OMB_Analyzing-Regulatory-Benefits-and-Costs.pdf

[34] American Society of Addiction Medicine. Public policy statement on the use of naloxone for the prevention of opioid overdose deaths. 2016. Accessed January 12, 2022. https://www.asam.org/docs/default-source/public-policy-statements/use-of-naloxone-for-the-prevention-of-opioid-overdose-deaths-final.pdf

[35] Carroll JJ, Green TC, Noonan RK. Evidence-based strategies for preventing opioid overdose: what’s working in the United States. National Center for Injury Prevention and Control, Centers for Disease Control and Prevention. 2018. Accessed January 12, 2022. https://www.cdc.gov/drugoverdose/pdf/pubs/2018-evidence-based-strategies.pdf

[36] Wood CA, Green L, La Manna A, . Balancing need and risk, supply and demand: developing a tool to prioritize naloxone distribution. Subst Abus. 2021;42(4):974-982. doi:10.1080/08897077.2021.1901174 33759727

[37] Weiner J, Murphy SM, Behrends C. Expanding access to naloxone: a review of distribution strategies. Leonard Davis Institute of Health Economics. May 2019. Accessed February 9, 2021. https://collections.nlm.nih.gov/catalog/nlm:nlmuid-101750194-pdf

[38] Kinnard EN, Bluthenthal RN, Kral AH, Wenger LD, Lambdin BH. The naloxone delivery cascade: identifying disparities in access to naloxone among people who inject drugs in Los Angeles and San Francisco, CA. Drug Alcohol Depend. 2021;225:108759. doi:10.1016/j.drugalcdep.2021.108759 34058540

[39] Madden EF, Qeadan F. Racial inequities in U.S. naloxone prescriptions. Subst Abus. 2020;41(2):232-244. doi:10.1080/08897077.2019.1686721 31718487

[40] Prevent Overdose RI. Get naloxone. Accessed July 22, 2021. https://preventoverdoseri.org/get-naloxone/

[41] Charest R, Novais A. Governor Daniel J. McKee’s Task Force on Overdose Prevention and Intervention. March 9, 2022. Accessed April 29, 2022. https://preventoverdoseri.org/wp-content/uploads/2022/03/March-2022-Task-Force-Master-PowerPoint-Final.pdf

[42] State of Rhode Island. Attorney General announces additional opioid settlements valued at more than $100 million against manufacturers Teva and Allergan. March 21, 2022. Accessed September 13, 2022. https://riag.ri.gov/press-releases/attorney-general-announces-additional-opioid-settlements-valued-more-100-million

[43] Harocopos A, Gibson BE, Saha N, . First 2 months of operation at first publicly recognized overdose prevention centers in US. JAMA Netw Open. 2022;5(7):e2222149. doi:10.1001/jamanetworkopen.2022.22149 35838672 PMC9287749

[44] Rubin R, Suran M. Supervised consumption sites—a tool for reducing risk of overdose deaths and infectious diseases in people who use illicit drugs. JAMA. 2022;327(16):1532-1534. doi:10.1001/jama.2022.4017 35385054

[45] Irvine MA, Buxton JA, Otterstatter M, . Distribution of take-home opioid antagonist kits during a synthetic opioid epidemic in British Columbia, Canada: a modelling study. Lancet Public Health. 2018;3(5):e218-e225. doi:10.1016/S2468-2667(18)30044-6 29678561

[46] Lewis CR, Vo HT, Fishman M. Intranasal naloxone and related strategies for opioid overdose intervention by nonmedical personnel: a review. Subst Abuse Rehabil. 2017;8:79-95. doi:10.2147/SAR.S101700 29066940 PMC5644601

[47] Fairley M, Humphreys K, Joyce VR, . Cost-effectiveness of treatments for opioid use disorder. JAMA Psychiatry. 2021;78(7):767-777. doi:10.1001/jamapsychiatry.2021.0247 33787832 PMC8014209

[48] Barocas JA, White LF, Wang J, . Estimated prevalence of opioid use disorder in Massachusetts, 2011-2015: a capture-recapture analysis. Am J Public Health. 2018;108(12):1675-1681. doi:10.2105/AJPH.2018.304673 30359112 PMC6236756

[49] Tobias S, Shapiro AM, Grant CJ, Patel P, Lysyshyn M, Ti L. Drug checking identifies counterfeit alprazolam tablets. Drug Alcohol Depend. 2021;218:108300. doi:10.1016/j.drugalcdep.2020.108300 33127185

